# HIV Envelope gp120 Activates LFA-1 on CD4 T-Lymphocytes and Increases Cell Susceptibility to LFA-1-Targeting Leukotoxin (LtxA)

**DOI:** 10.1371/journal.pone.0023202

**Published:** 2011-08-05

**Authors:** Catarina E. Hioe, Michael Tuen, Gaia Vasiliver-Shamis, Yelina Alvarez, Kathleen C. Prins, Sagarika Banerjee, Arthur Nádas, Michael W. Cho, Michael L. Dustin, Scott C. Kachlany

**Affiliations:** 1 Department of Pathology, New York University School of Medicine, and Veterans Affairs New York Harbor Healthcare System, Manhattan Campus, New York, New York, United States of America; 2 Program in Molecular Pathogenesis, Marty and Helen Kimmel Center for Biology and Medicine, Skirball Institute for Biomolecular Medicine, New York University School of Medicine, New York, New York, United States of America; 3 Department of Environmental Medicine, New York University School of Medicine, New York, New York, United States of America; 4 Department of Biomedical Sciences, College of Veterinary Medicine, Iowa State University, Ames, Iowa, United States of America; 5 Department of Oral Biology, New Jersey Dental School, University of Medicine and Dentistry of New Jersey, Newark, New Jersey, United States of America; 6 Actinobac Biomed, Incorporated, North Brunswick, New Jersey, United States of America; Mayo Clinic, United States of America

## Abstract

The cellular adhesion molecule LFA-1 and its ICAM-1 ligand play an important role in promoting HIV-1 infectivity and transmission. These molecules are present on the envelope of HIV-1 virions and are integral components of the HIV virological synapse. However, cellular activation is required to convert LFA-1 to the active conformation that has high affinity binding for ICAM-1. This study evaluates whether such activation can be induced by HIV itself. The data show that HIV-1 gp120 was sufficient to trigger LFA-1 activation in fully quiescent naïve CD4 T cells in a CD4-dependent manner, and these CD4 T cells became more susceptible to killing by LtxA, a bacterial leukotoxin that preferentially targets leukocytes expressing high levels of the active LFA-1. Moreover, virus p24-expressing CD4 T cells in the peripheral blood of HIV-infected subjects were found to have higher levels of surface LFA-1, and LtxA treatment led to significant reduction of the viral DNA burden. These results demonstrate for the first time the ability of HIV to directly induce LFA-1 activation on CD4 T cells. Although LFA-1 activation may enhance HIV infectivity and transmission, it also renders the cells more susceptible to an LFA-1-targeting bacterial toxin, which may be harnessed as a novel therapeutic strategy to deplete virus reservoir in HIV-infected individuals.

## Introduction

Efficient progression of the different steps in human immunodeficiency virus (HIV) replication, from virus-cell attachment to virus progeny production, is tightly linked to the activation state of the host CD4 T cells. T cell activation impacts on the initial HIV interaction with the target CD4 T cells through the involvement of adhesion molecule leukocyte functon antigen-1 (LFA-1) and its inter-cellular adhesion molecule-1 (ICAM-1) ligand [1], [2]. LFA-1 and ICAM-1 are incorporated into the envelope of HIV-1 virions budding from activated primary CD4 T cells that support productive virus replication [3], [4]. HIV-1 virions bearing ICAM-1 are more infectious than their ICAM-1-negative counterparts, due to enhanced virus binding to LFA-1-bearing target cells [5], [6]. Therefore, LFA-1 expression on target cells increases susceptibility to HIV-1 infection via cell-free virions [1], [7], [8]. LFA-1 and ICAM-1 also play a critical role in cell-cell transmission as these adhesion molecules are integral components of the HIV-1 virological synapse [9], [10], which serves as a major mode for HIV-1 transmission from cell to cell. Furthermore, ICAM-1 expression on HIV-1 virions alone or in combination with LFA-1 on the target cells increases HIV-1 resistance to neutralizing antibodies [2], [11]–[13]. One should note, however, that LFA-1 must first be activated to mediate its function as the receptor for ICAM-1 [14]. The inactive form binds poorly to its ligand, but upon T cell activation, LFA-1 undergoes conformational changes that increase its affinity for ICAM-1. Under physiologic condition, LFA-1 activation results from TCR engagement of the specific peptide-MHC complex and inside-out signaling induced as part of the canonical TCR activation signals [15]. Activation of host T cells is also critical for the steps of virus life cycle subsequent to virus attachment and entry. Quiescent CD4 T cells at G_0/1a_ phase are refractory to infection, due to the slow kinetics of the early steps of the virus replication including the inefficient reverse transcription, and cellular activation that propels the cells into the G1_b_ phase or beyond is needed for virus infection to proceed [16]. After the integrated provirus is established, virus gene expression is again regulated by cellular activation via the participation of cellular nuclear factors NF-kB and/or NFAT [17]. The activation of these transcription factors is a downstream 2 event of the TCR-induced signaling cascade; NF-kB is activated via the diacylglycerol/protein kinase C pathway, while NFAT activation is induced by the IP3/calcineurin pathway [18].

Considering the importance of host T cell activation in HIV replication and the advantageous contributions of LFA-1-ICAM-1 interactions to HIV-1 infectivity, we postulate that HIV via its envelope protein gp120 exploits the T cell activation mechanism and triggers LFA-1 activation in order to promote its infectivity, replication, transmission, and resistance from anti-viral immunity. HIV gp120 binding to CD4 and/or the chemokine receptor has been shown to trigger activation of different signaling pathways and virus replication in the absence of conventional activation markers [19]–[24]. Our previous studies have also demonstrated that the interaction of surface-bound gp120 with CD4 on activated primary CD4 T cells induces activation of some components of the T cell signaling machinery, albeit without stimulating full T cell activation [25]. Importantly, this cellular activation is sufficient to cause rearrangement of LFA-1-ICAM-1 interactions leading to the formation of an adhesive ring resembling a peripheral supramolecular activation cluster of the T cell immunological synapse [25], [26]. LFA-1 activation has also been reported to result from gp120 engagement of the active form of the integrin α4β7 on retinoic acid-treated CD4 T cells [27]. Nevertheless, the capacity of gp120 to fully activate LFA-1 from its inactive state has not been evaluated directly.

In the present study, we investigated gp120-induced LFA-1 activation by monitoring the interaction of naïve resting CD4 T cells that express LFA-1 in its inactive form with ICAM-1 in the presence of gp120 bound onto laterally mobile planar bilayers. LFA-1 expression was also monitored on CD4 T cells with active HIV infection from the peripheral blood of HIV-infected subjects. We further confirmed virus-associated LFA-1 activation by utilizing a leukotoxin (LtxA) from an oral bacterium *Aggregatibacter actinomycetemcomitans* that is known to preferentially target cells expressing the active form of LFA-1 [28]. LtxA is effective against LFA-1-expressing leukocytes of humans and Old World primates. A single intravenous administration of LtxA in a healthy uninfected rhesus macaque has been shown to cause a substantial but transient drop of white blood cell counts in the peripheral blood for ∼12 hrs without any effects on red blood cell, platelet, and hemoglobin values and without any signs of liver toxicity or kidney dysfunction [28]. In addition, LtxA was highly effective at treating leukemia in a humanized SCID mouse model [28]. Nevertheless, the effects of LtxA on cellular reservoirs of HIV-1 remain to be determined. This study demonstrates that the presence of activated LFA-1 was characteristic of CD4 T cells which were exposed to HIV envelope in vitro and CD4 T cells that supported HIV replication in the peripheral blood of HIV-1 infected subjects. These virus-associated CD4 T cells were sensitive to killing by LFA-1-specific LtxA. This study presents initial data for the potential development of a bacterial leukotoxin as a therapeutic reagent to deplete host cells recently exposed to or already infected with HIV to reduce the overall viral burden in HIV-infected individuals.

## Results

### The interaction of CD4 T cells with HIV-1 gp120 on bilayers triggers LFA-1 activation and supramolecular re-organization

The virus envelope glycoprotein gp120 has been shown to induce CD4 T cell activation [19], [22]–[25], but there has not been any direct evidence of gp120 to activate LFA-1 on completely quiescent CD4 T cells. In a recent study, we demonstrated that the interaction of CD4 T cells with gp120 on a glass-supported laterally-mobile planar bilayer causes phosphorylation of many signaling molecules involved in TCR-induced activation, albeit in the absence of full T cell activation [25]. However, because the CD4 T cells tested in that study were already activated with anti-CD3 and anti-CD28 antibodies, their LFA-1 was in the high affinity state and therefore the capacity of gp120 to trigger LFA-1 activation could not be studied. To address this question, we analyzed the interaction of resting naïve CD4 T cells obtained ex vivo from the peripheral blood of healthy HIV-seronegative donors with gp120 and ICAM-1 on glass-supported planar lipid bilayers. The bilayers served as an experimental model mimicking the virion surface or the infected cell surface. To discern changes in cellular morphology and molecular organization, the bilayers were loaded with Alexa Fluor 488-labeled gp120 and Cy5-labeled ICAM-1. For comparison, bilayers were also prepared with Cy5-labeled ICAM-1 alone. After the cells were added onto the bilayers, live images were acquired for up to 1 hr using multicolor total internal reflectance fluorescent (TIRF) microscopy.

Naïve peripheral CD4 T cells express LFA-1 in the inactive states with low affinity for ICAM-1. Indeed, naïve CD4 T cells enriched from PBMCs of HIV-seronegative donors were CD4+, CD3+, CD45RA+, CD45R0- and were not stained with mAb NKI-L16 (Figure S1), which binds to an epitope on the active-state, extended conformation of the LFA-1 α subunit [29]. By contrast, this same mAb stained 11.6% of cells in the unfractionated PBMCs.

**Figure 1:**
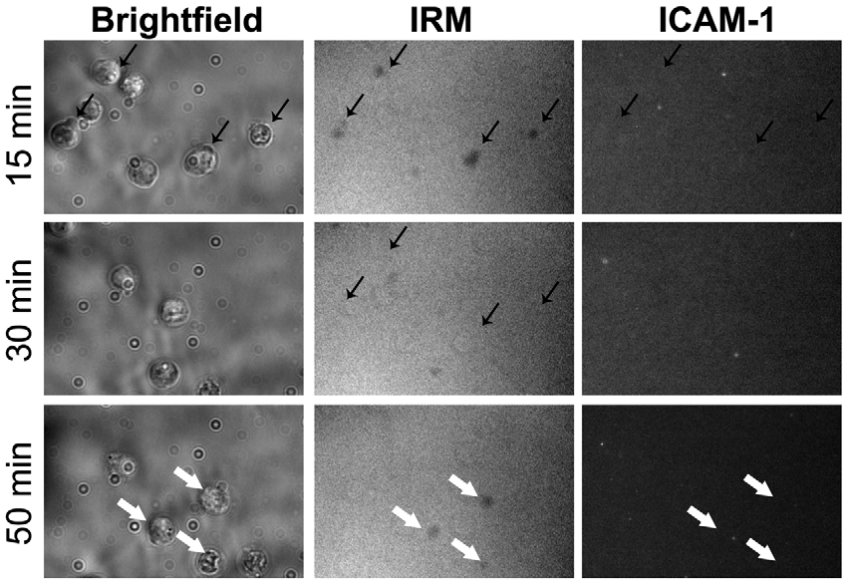
Naïve CD4 T cells do not form stable interaction with the ICAM-1 bilayer. The naïve CD4 T cells were injected onto a bilayer containing only ICAM-1 and monitored over one hour for the presence of cells (bright-field panels), contact with the bilayer (interference reflection microscopy (IRM) panels) and contact with ICAM-1 (ICAM-1 panel). The same representative field is shown at the indicated time points. Thin black arrows show the cells that interacted transiently with the bilayers at 15 min but had no ICAM-1 accumulation and migrated by 30 min. Three new cells interacting with the bilayer at 50 min were also observed and indicated by thick white arrows; again these cells made no ICAM-1 contact and did not induce any ICAM-1 aggregation.

**Figure 2:**
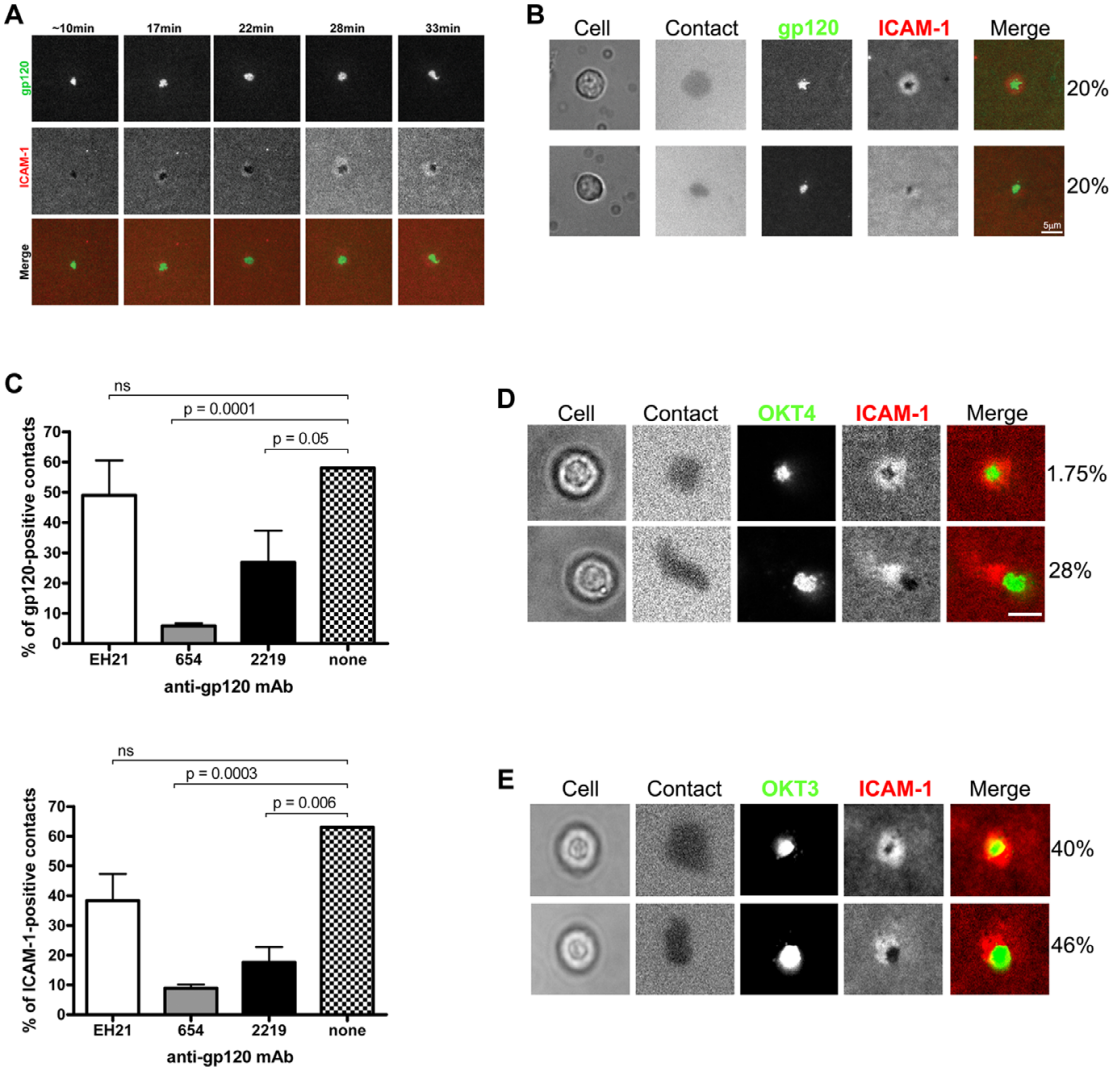
HIV gp120 interaction with quiescent naïve CD4 T cells triggers LFA-1 activation and supramolecular rearrangement. (A) Naïve CD4 T cells establish LFA-1-ICAM-1 interaction upon gp120 binding. The naïve CD4 T cells purified by negative selection from ex vivo HIV-seronegative PBMCs were introduced to a bilayer containing Alexa Fluor 488-labeled gp120 and Cy5-labeled ICAM-1, and images were acquired over an hour. Images from one representative cell to show the dynamics of cell interaction with gp120 and ICAM-1 over time are presented. Top panels show gp120 contact the cell made at the indicated time points, middle panels show ICAM-1 contact, and bottom panels display merged images (gp120 in green, ICAM-1 in red). (B) Morphology of ICAM-1 contact areas made upon the interaction of naïve CD4 T cells with gp120 and ICAM-1 on the bilayers. Images of representative cells and the percentages of cells that form symmetrical (top panel) or asymmetrical (bottom panel) ICAM-1 rings are shown. gp120 was added onto the bilayers at a density of 200 to 250 molecules/µm^2^. (C) Naïve T cells were added to the bilayers in the presence of an anti-gp120 mAb that blocks gp120-CD4 interaction (654), an anti-V3 mAb that interferes with gp120 binding to the chemokine receptor (2219), or a mAb to the N-terminus of gp120 (EH21) that does not affect gp120 interaction with its receptors. The percentages of cells making gp120-positive (left) and ICAM-1-positive (right) contacts out of the total number of cells seen in the fields were calculated. The MAbs were used at 20 µg/ml. The graphs represent the averages +/- SEM of three independent experiments. Statistical analysis was done by one-sided Student's t test. (D and E) Morphology of ICAM-1 contact areas made upon the interaction of naïve CD4 T cells with bilayers containing ICAM-1 and monoclonal antibodies to CD4 (OKT4) or CD3 (OKT3). The percentages of cells that form symmetrical (top panel) or asymmetrical (bottom panel) ICAM-1 rings are shown for comparison with those observed in gp120 + ICAM-1 bilayers (B). The densities of OKT4 and OKT3 on the bilayer were 250 molecules/µm^2^.

On bilayers containing ICAM-1 alone, these naïve cells rarely formed contact with the bilayers and if they did, the contact was transient and did not cause any ICAM-1 accumulation (Figure 1). However, when these cells were introduced onto bilayers containing gp120 and ICAM-1, cells established gp120 contacts within the first 5–10 min and 10 min later started forming ICAM-1 contact (Figure 2A). About 40% of the cells established stable LFA-1-ICAM-1-mediated adhesion (Figure 2B). In addition, supramolecular rearrangements of both gp120 and ICAM-1 were observed: gp120 accumulated into a central cluster and ICAM-1 assembled into a ring, which was either symmetrical (for 20% of the cells; Figure 2B top) or asymmetrical (for another 20%; Figure 2B bottom), around the central gp120 cluster. Once formed, the morphology was maintained for the duration of the experiment (1 hr). The remaining cells contacted gp120 but did not accumulate ICAM-1 or did not make stable ICAM-1 contact (not shown). The LFA-1-ICAM-1 interaction and rearrangement were triggered specifically by gp120 binding to its receptors on the T cell surface, since pre-treatment with mAbs to the CD4-binding site (654) substantially reduced not only the numbers of cells forming gp120 contact (Figure 2C, left graph), but also ICAM-1 contact (Figure 2C, right graph). MAb 2219 against the V3 loop, which is involved in binding the chemokine receptor, also decreased both gp120 and ICAM-1 contacts, although it was not as effective as the anti-CD4 binding site mAb. A mAb to the N-terminus of gp120 (EH21) which does not participate in CD4 or the chemokine receptor binding had no effect as both gp120 and ICAM-1 contacts were comparable to those of untreated controls.

To determine whether engagement of CD4 alone is sufficient to induce LFA-1 activation, we introduced naïve CD4 T cells onto bilayers containing an anti-CD4 mAb and ICAM-1. About 28% of the cells established contact with the mAb and ICAM-1, but ICAM-1 accumulation assembled mainly into an asymmetrical ring (Figure 2D). Only 1.75% of the cells formed symmetrical ICAM-1 rings, which were transient, lasting only two minutes. The majority of the cells (68%) made contact only with the anti-CD4 mAb and had no ICAM-1 accumulation. The remaining cells had neither ICAM-1 nor anti-CD4 mAb contacts. For comparison, LFA-1 activation of naïve CD4 T cells as a result of the physiologic T cell receptor (TCR) engagement was also evaluated by monitoring the interaction of the naïve cells with bilayers containing anti-CD3 mAb and ICAM-1. The vast majority of the cells displayed CD3 and ICAM-1 supramolecular arrangement; 40% formed the conventional immunological synapse with a central CD3 cluster and a symmetrical ICAM-1 ring, and 46% had an asymmetrical ICAM-1 ring (Figure 2E). Importantly, these synapses were very stable, lasting throughout the entire experiment (45 min), similar to those induced upon interaction with gp120 but not with the anti-CD4 mAb. These data indicate that gp120 binding to its receptor CD4 triggers LFA-1 activation and supramolecular rearrangement, as revealed by formation of synapses with stable LFA-1-ICAM-1 interactions. However, this LFA-1 activation is not as robust as that triggered via the T cell receptor. CD4 engagement by antibody also weakly induces LFA-1 activation, but generates a distinct pattern of LFA-1-ICAM-1 supramolecular organization. Hence, the effect of gp120 on naïve CD4 T cells is unique and cannot be fully mimicked by an anti-CD4 mAb.

### CD4 T cells exposed to surface-bound gp120 are more susceptible to killing by the LFA-1-targeting bacterial leukotoxin (LtxA)

**Figure 3:**
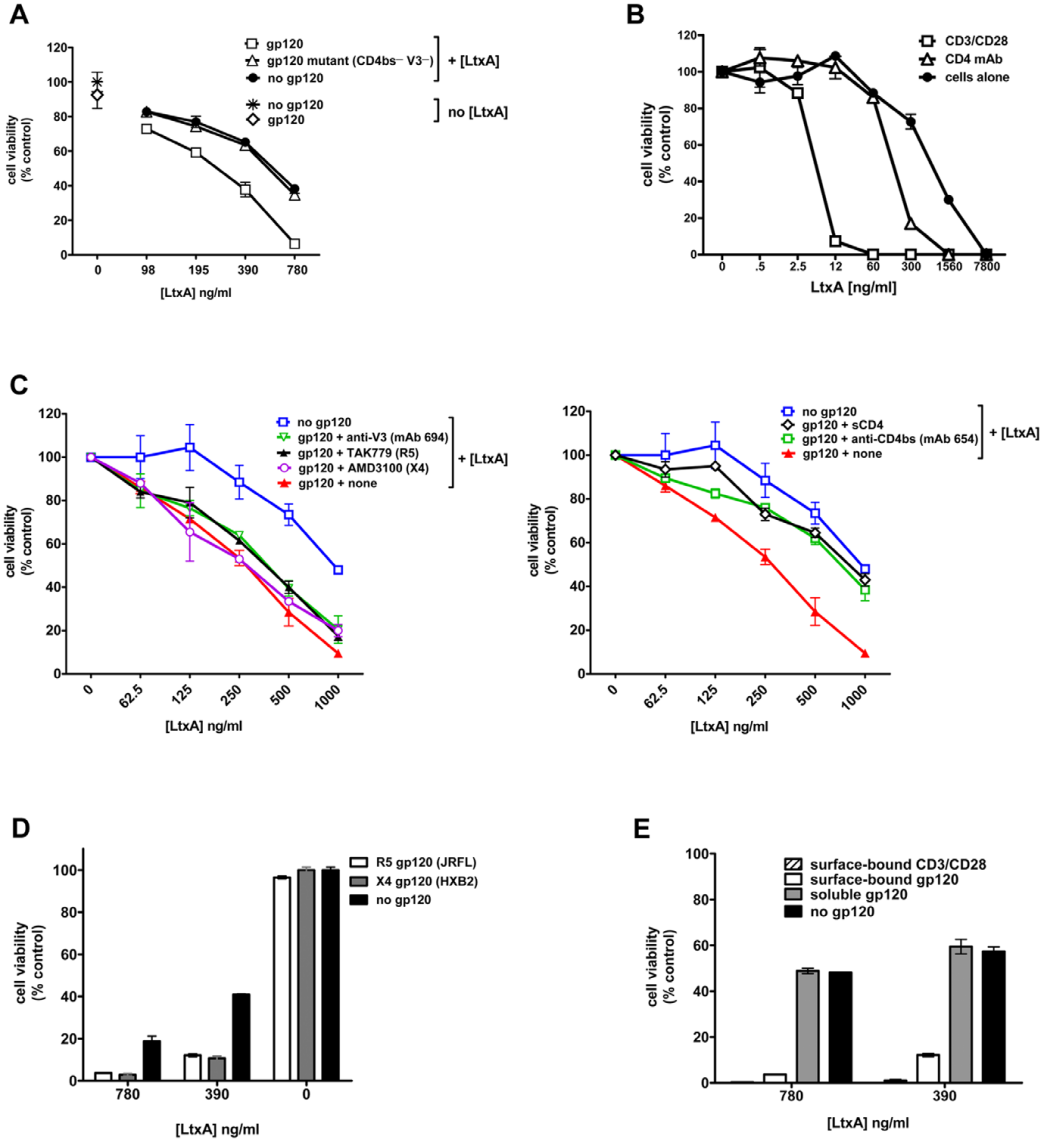
The interaction of quiescent CD4 T cells with surface-bound gp120 renders the cells more susceptible to leukotoxin LtxA. (A) CD4 T cells purified ex vivo from HIV-seronegative PBMCs were incubated on wells coated with 10 µg/ml wild type gp120 (JRFL), mutant gp120 (JRFL) lacking CD4-binding site and V3, or no gp120 and then treated with titrated amounts of LtxA. (B) For comparison, CD4 T cells were also incubated on wells coated with anti-CD3 and anti-CD28 antibodies (5 µg/ml each) or anti-CD4 antibody (10 µg/ml) and treated with the designated concentrations of LtxA. (C) CD4 T cells were incubated on gp120-coated wells in the presence of monoclonal antibodies (mAb) against the CD4-binding site (anti-CD4bs) or the V3 loop of gp120, soluble CD4 (sCD4), or the chemokine receptor antagonists, and then treated with titrated amounts of LtxA. (D) R5 gp120 (JRFL) or X4 gp120 (HXB2) was used to coat the wells, incubated with CD4 T cells, and tested for induction of LtxA-mediated killing. In A, C, and D, the gp120 proteins were coated on microtiter wells at 10 µg/ml. (E) CD4 T cells were treated with soluble gp120 (10 µg/ml), anti-CD3/anti-CD28 coated on wells, or gp120 coated on wells, prior to addition of LtxA. After incubation with LtxA for 20 hrs, the cell viability was measured based on cellular ATP. Data from one of two or more representative experiments are shown. The mean and standard deviation values from duplicate wells are presented.

LFA-1 activation is triggered by gp120 upon binding to CD4 T cells therefore, we surmise that these cells should become highly susceptible to LtxA, a bacterial leukotoxin that is known to preferentially kill leukocytes with high levels of the activated form of LFA-1 [28]. To examine this idea, we incubated resting CD4 T cells from healthy uninfected donors on tissue culture wells coated with or without gp120 and then treated them with different concentrations of LtxA. After 20 hrs, cell viability was determined by measurement of cellular ATP. A mutant gp120 protein lacking the ability to bind CD4 and the chemokine receptors (CD4bs- V3-) was tested as a control. Data in Figure 3A shows that CD4 T cells interacting with gp120 were more susceptible to LtxA than cells incubated with the mutated gp120 or no gp120. The increased cell death was apparent at different concentrations of LtxA. The 50% effective dose (ED50) of LtxA against gp120-treated CD4 T cells was 263 ng/ml, while the ED50 of LtxA against untreated cells and mutant gp120-treated cells were 577 and 540 ng/ml, respectively. In other experiments with cells from two different donors, ED50 of LtxA against gp120-treated CD4 T cells ranged from 150 to 276 ng/ml (mean and standard deviation, 213 +/− 89 ng/ml). Treatment with gp120 without LtxA had no significant effect on the CD4 T cell viability (Figure 3A). The enhanced susceptibility to LtxA as a result of gp120 interaction was modest when compared with canonical T cell activation by surface-bound anti-CD3 and anti-CD28 antibodies (Figure 3B). Stimulation with the anti-CD3+CD28 antibodies increased cell susceptibility to LtxA by >100 fold (ED50 of 5 ng/ml). This confirms our previous data showing the strong preference of LtxA for active LFA-1-expressing T cells [28].

To verify the specificity of the gp120 effect and assess the molecular interactions involved, we first measured LtxA susceptibility of naïve CD4 T cells treated with the anti-CD4 antibody. The data show that naïve CD4 T cells interacting with anti-CD4 antibody coated on wells also became more susceptible to LtxA (Figure 3B), indicating the contribution of CD4 engagement in LFA-1 activation. The mean ED50 of LtxA against anti-CD4-antibody-treated cells was 180 ng/ml (standard deviation, 57 ng/ml), comparable to that against gp120-treated cells. Subsequently, we used mAbs against different gp120 epitopes or small molecules that block gp120 interaction with CD4 or the chemokine receptors and measured their effects on LtxA susceptibility of gp120-treated cells. The enhanced susceptibility to LtxA was abrogated by mAb 654 or soluble CD4, both of which blocks gp120-CD4 interaction, while mAb against the V3 loop of gp120 (694) or chemokine receptor antagonists (AMD3100 [CXCR4-specific] or TAK779 [CCR5-specific]) had minimal effects (Figure 3C). These results consistently demonstrate the importance of gp120 binding to CD4, but not the chemokine receptor, in triggering LFA-1 activation.

We further demonstrate that both R5 and X4 gp120 were able to enhance susceptibility of naïve CD4 T cells to LtxA (Figure 3D). In the absence of LtxA, R5 or X4 gp120 did not reduce CD4 T cell viability. However, the enhanced killing was induced only when the cells interacted with gp120 bound on the well surface; soluble gp120 did not have the same effect (Figure 3E), indicating that gp120-mediated crosslinking of CD4 is essential for LFA-1 activation that renders the CD4 T cells more susceptible to killing by LFA-1-specific LtxA. CD4 T cells activated by surface-bound anti-CD3 and anti-CD28 antibodies were used as a control, and nearly 100% of these cells were killed by LtxA at the concentrations tested (Figure 3E). These data demonstrate that gp120 binding to quiescent naïve CD4 T cells renders the cells more susceptible to LtxA due to LFA-1 activation as a result of CD4 cross-linking, although the gp120-induced activity is not as potent as that triggered by TCR engagement.

### Viral p24-producing CD4 T cells in the peripheral blood of HIV-infected subjects display higher levels of total and active LFA-1

**Figure 4:**
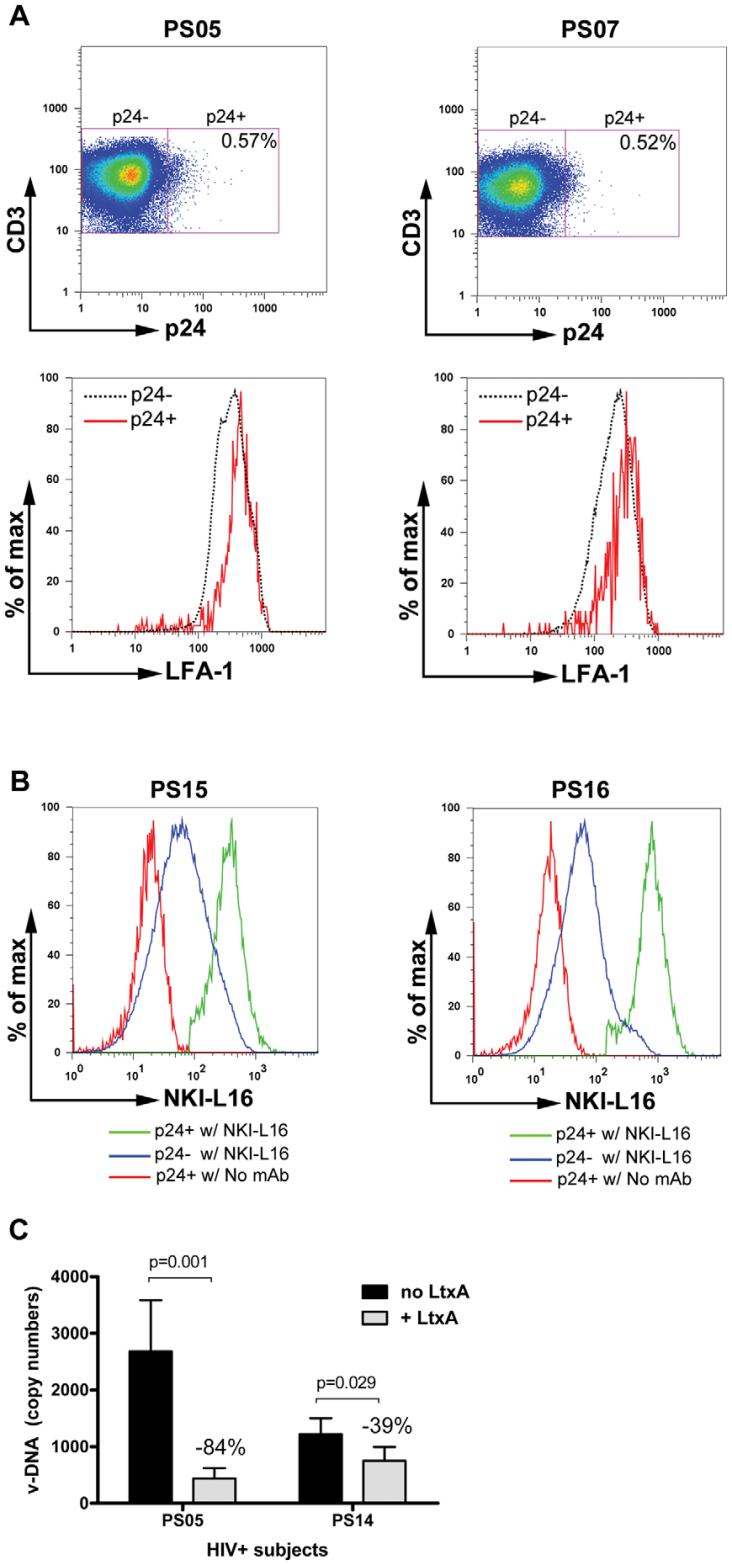
(A) CD4 T cells bearing HIV p24 antigen express higher levels of total LFA-1 on the surface. Ex vivo PBMCs from two viremic HIV-infected subjects (PS05 with 38,165 vRNA copies/ml and PS07 with 35,201 vRNA copies/ml) were stained with mAbs for surface expression of CD3, CD8, and total LFA-1, as well as for intracellular p24 antigen after permeabilization. The cells were subjected to flow cytometric analyses, and the data analyzed by the FlowJo software. The dot plots (top) show the percentages of p24+ cells among CD4 T cells (CD3+ CD8-) from subjects PS05 and PS07. The p24+ gating were determined based on p24 staining of HIV-seronegative PBMCs (Fig. S3). The histograms (bottom) compare LFA-1 expression on p24+ and p24- CD4 T cells; the mean fluorescence intensity (mfi) for p24+ and p24- CD4 T cells are 462 and 376 for PS05, and 311 and 228 for PS07. (B) Expression of active LFA-1 on p24+ versus p24- CD4 T cells. PBMCs from viremic HIV-infected subjects (PS15 with 5,075 vRNA copies/ml and PS16 with 7,077 vRNA copies/ml) were stained with mAb NKI-L16 and secondary fluorescent anti-mouse antibodies, followed with direct staining with fluorescent mAbs to CD3, CD8 and p24. The histograms compare mAb NKI-L16 staining on p24+ and p24- CD4 T cells; the mfi for p24+ and p24- cells are 405 and 95 for PS15 and 835 and 86 for PS16. p24+ CD4 T cells treated with the secondary antibody (no NKI-L16) were also shown for control (mfi = 19 and 18). (C) Reduction of viral DNA in HIV-infected PBMCs due to LtxA cytotoxicity. PBMCs from two viremic HIV-infected subjects (PS05 with 38,165 vRNA copies/ml and CD4 count of 814 and PS14 with 21,815 vRNA copies/ml and CD4 count of 494) were treated with LtxA (7.8 µg/ml) for 20 hrs. The viral DNA were quantified by real time PCR with the specific primers. Averages and standard deviation from epeat experiments are presented. Statistical analysis was done by one-sided Student's t test.

To evaluate LFA-1 expression on HIV-infected CD4 T cells in the peripheral blood, we analyzed ex vivo PBMCs from two viremic untreated HIV-infected subjects (PS05 and PS07) who were asymptomatic and had CD4 counts of >450. Virus-infected cells were detected by flow cytometry following intracellular staining with an anti-p24 monoclonal antibody and surface staining with monoclonal antibodies to the phenotype markers (CD3 and CD8) and LFA-1. The dot plots in Figure 4A shows that 0.57% and 0.52% of p24+ CD3+ CD8- T cells were detected in subjects PS05 and PS07, respectively. The p24+ gating is based on the p24 staining of HIV-seronegative PBMCs tested in parallel in each assay (Figure S2-A). When the total LFA-1 expression of p24+ and p24- cells was compared, higher levels were detected on the p24+ cell population than on the p24- cell population from the same subjects. The higher LFA-1 expression levels were specific as no increase in CD3 expression was observed on p24+ cells as compared to p24- cells (Figure S2-B). PBMCs from two additional viremic HIV-infected subjects (PS15 and PS16) were also stained with mAb NKI-L16, which is specific for the active LFA-1 α subunit [29]. The data in Figure 4B show that p24+ CD4 T cells indeed expressed higher levels of active LFA-1 than p24- cells, indicative of the activated state of HIV-infected CD4 T cells producing viral p24 antigens in the peripheral blood.

### LtxA treatment of HIV-infected PBMCs decreases HIV DNA levels

Considering that CD4 T cells supporting active HIV infection have higher levels of surface LFA-1 expression and that LtxA has been shown to preferentially target activated CD4 T cells expressing higher levels of LFA-1, we examined whether LtxA treatment can target the infected CD4 T cells and reduce the levels of viral DNA in the PBMCs of HIV-infected individuals. PBMCs from two viremic HIV-seropositive subjects (PS05 and PS14) were treated with a single dose of LtxA (7.8 µg/ml) for 20 hrs, and the amounts of viral DNA and β-actin quantified by real time PCR. The data in Figure 4C show that 2682 and 1223 copies of viral DNA were initially detected in PBMCs of subjects PS05 (∼2.5×10^5^ cells) and PS014 (∼1.2×10^5^ cells), respectively, and LtxA treatment reduced viral DNA by 84% and 39%. The reduction of viral DNA was accompanied by comparable reduction in β-actin copy numbers (Figure S3). Since virus infected cells constituted only ∼0.5% of total peripheral CD4 T cells, these data indicate that the cells targeted by LtxA are likely to include uninfected bystanders as well as HIV infected cells. Overall, this study indicates that LtxA may be an effective compound for removing cells bearing HIV-1 and reducing viral DNA loads but LtxA can also affect bystander uninfected cells, although the biologic consequences of such depletion remains to be investigated.

## Discussion

Unlike CD4 and the chemokine receptors, LFA-1 and ICAM-1 are not absolutely required for HIV infection. However, the important role of these cellular adhesion molecules in enhancing virus infectivity and transmission by promoting virus-cell attachment and cell-cell adhesion has been consistently demonstrated [1], [5]–[7]. In this study we further show that HIV-1 itself is capable of stimulating LFA-1 and converting it to an active conformation that allows binding to ICAM-1 with high affinity. The functional evidence was provided in the glass-supported planar bilayer system in which we observed that the interaction of HIV-1 envelope gp120 with fully quiescent, naïve CD4 T cells enabled stable interaction of LFA-1 on the T cell surface with ICAM-1 on the bilayer. In the absence of gp120, naïve T cells rarely adhered to ICAM-1. Interestingly, gp120 binding to the naïve CD4 T cells also triggered the rearrangement of LFA-1-ICAM-1 interactions creating an adhesive ring around a central gp120 accumulation, reminiscent of the pSMAC and c-SMAC structures commonly found in the T cell receptor-induced immunological synapse. The same patterns of gp120 and ICAM-1 supramolecular structures were observed when the activated CD4 T cells interacted with gp120 on the planar bilayers [25], [26]. The ability of gp120 to activate LFA-1 was further evidenced by the increased sensitivity of quiescent T cells upon interaction with surface-bound gp120 to LtxA, a bacterial leukotoxin known to preferentially kill leukocytes bearing the active form of LFA-1 [28]. gp120-induced LFA-1 activation is likely to promote the formation of the HIV virological synapse, which is a structure at the cell-cell junction created by the tight adhesion of LFA-1-ICAM-1 [9], [10], [25], [26], [30], [31] The formation of a symmetrical LFA-1-ICAM-1 ring in particular indicates that cell migration is arrested [26] to allow formation of a stable cell-cell conjugate which facilitates efficient HIV transfer from infected cells to new target cells in a protected compact environment. Beyond the initial virus-cell interaction, it remains unclear to what extent gp120-induced LFA-1 activation contributes to a heighten state of immune activation commonly observed during chronic HIV infection.

The molecular mechanisms for gp120-induced LFA-1 activation are not fully understood. The data presented here show that gp120 binding to CD4 is crucial for LFA-1 activation, as antibody or other compounds that block gp120-CD4 interaction prevented cell contact with ICAM-1 and nullified the enhanced susceptibility of the cells to LtxA. The involvement of the chemokine receptor, on the other hand, is still unclear. The antibody against V3, which is critical for gp120 interaction with the chemokine receptor, caused only partial inhibition of ICAM-1 contact. The anti-V3 antibody and chemokine receptor antagonists had minimal effects on LtxA susceptibility of cells that interacted with gp120. In previous studies, gp120 binding to CD4 on activated CD4 T cells also induced formation of supramolecular rearrangement characterized with a central gp120 cluster and a peripheral LFA/ICAM ring [26], and this supramolecular rearrangement could be blocked by antibodies to the CD4-binding site but not by anti-V3 antibodies or the chemokine receptor antagonists. The gp120 protein used in the planar bilayer system is from the dual tropic strain of HIV-1 DH12, while both R5 gp120 (JRFL) and X4 gp120 (IIIB) proteins were found to be comparable in inducing enhanced sensitivity to LtxA. Considering that resting and naive T cells express mainly CXCR4 and no or very low levels of CCR5, the ability of R5 gp120 to activate LFA-1 suggests that gp120 binding to CD4 is the key factor that mediates this activity.

Consistent with this idea, CD4 cross-linking by antibodies was able to activate LFA-1 to form ICAM-1 contacts on the bilayers and cause a detectable increase in the naïve T cell susceptibility to LtxA. Nevertheless, the levels of LFA-1 activation detected in the two assays were not identical. While gp120 induced higher numbers of CD4 T cells forming LFA-1/ICAM-1 interaction on the bilayers than the anti-CD4 antibody OKT4, gp120 and OKT4 increased LtxA susceptibility to comparable extents. This disparity may be attributed to differences in duration and sensitivity of the two assays. The LtxA-mediated killing was measured after an overnight incubation and may not detect differences in the kinetics and the early stages of LFA-1 activation triggered by gp120 as compared to OKT4. The bilayer experiments also provided live images showing that the ability of gp120 to induce formation of the more stable LFA-1-ICAM-1 adhesion rings was not fully replicated by an anti-CD4 antibody. Our previous studies showed that gp120 binding to CD4 T cells triggers intracellular signaling events that initiate from activation of Src family tyrosine kinase Lck and cascade toward LAT and PLC-γ [25]. Interestingly, antibodies that cross-link CD4 also stimulate Lck phosphorylation, albeit with a spatio-temporal pattern distinct from that triggered by gp120 [32], [33]. All together, these data indicate that gp120-CD4 interaction may elicit LFA-1 activation via intracellular signaling unique from that induced by the anti-CD4 antibody.

Under physiologic conditions, LFA-1 activation is triggered in naïve T cells following TCR recognition of the cognate peptide-MHC complexes in conjunction with costimulation from CD4 and CD28. TCR clustering rapidly recruits CD4-associated Lck, which becomes phosphorylated transiently and initiates phosphorylation ITAMs of the TCR-CD3 complex [18]. ZAP70 and LAT are then brought into the TCR-CD3 clusters, phosphorylated, and in turn recruit SLP76-Nck-Vav complex, PLC-γ, PI3K, and ITK. These TCR-proximal signaling events generate the second messengers that propagate the signals downstream to activate transcription factors such as NFAT and NFκB resulting in upregulation of gene expression. LFA-1 activation is triggered via the so-called “inside out” signaling pathway that starts from Vav at the LAT complex and propagates through PKC, ADAP, and SKAP55 to the integrin-associated signaling complex that includes PKD, Rap1, and talin, and then to RAPL and RIAM to result in increased adhesion of the LFA-1 ectodomain for its ICAM-1 ligand [15]. It is not known if the same LFA-1 activation pathway is triggered by gp120; however, CD4 T cell interaction with gp120 on the planar bilayers has been shown to cause recruitment and phosphorylation of Lck and downstream signaling molecules CD3ζ, ZAP70, LAT, SLP76 and PLCγ [25]. Activation of these signaling molecules indicates that gp120-induced signaling events are likely transduced downstream to the LAT/SLP76 activation complex to reach Vav, although the recruitment and activation of Vav have not been directly demonstrated. Nevertheless, the activity of LFA-1-targeting LtxA against CD4 T cells exposed to gp120 is much less potent than that against CD4 T cells fully activated by anti-CD3/CD28 antibodies, indicating that gp120-induced LFA-1 activation is dissimilar either qualitatively or quantitatively from that by the T cell receptor-induced stimuli. This observation is consistent with previous data showing that gp120 induces incomplete signaling that does not propagate beyond PLC-γ and fails to trigger Ca^2+^ flux [25].

While gp120-induced LFA-1 activation may be beneficial for the virus, we may also be able to take advantage of this activity as an anti-viral strategy. Hence, we present here for the first time the activity of LFA-1-targeting leukotoxin LtxA against CD4 T cells recently exposed to the HIV envelope and CD4 T cells supporting active HIV infection. Both cell types display activated phenotypes with enhanced levels of LFA-1 on the cell surface and thus are more susceptible to killing by LtxA. Importantly, the enhanced susceptibility to LtxA is exhibited by CD4 T cells interacting with surface-bound gp120 and not by cells simply exposed to soluble gp120 monomers that may be shed from the virus. Because LtxA targets an invariant cellular protein LFA-1 [28], the activity of LtxA is not affected by the tremendous genetic, biologic, and antigenic variation of HIV-1. Therefore, LtxA-based approach to eradicate HIV-infected cells differs from previously reported strategies of utilizing toxin-conjugated CD4 or antibodies that target productively infected cells via the virus envelope antigens [34], [35], which are not only highly variable but are also occluded to various degrees in the vast majority of HIV-1 clinical isolates. This strategy is also different from previously reported studies of utilizing LFA-1 antagonists that are aimed to reduce the efficiency of virus-cell interaction and suppress virus transmission [36]. LtxA kills activated CD4 T cells by an apoptotic mechanism as demonstrated by Annexin V upregulation [37]. LtxA may be administered in a short course along with ART. Previous studies demonstrate that three doses of LtxA to leukemic mice were sufficient to eliminate the malignancy and result in long-term disease-free progression [28]. The short-course administration of LtxA should reduce the risk of severe immunosuppression as reported with the clinical use of Efalizumab, a monoclonal antibody against the CD11a subunit of LFA-1 for treatment of psoriasis [38]. While ART successfully reduces viremia to <50 copies/ml, the virus often still replicates at low levels in infected CD4 T cells and other cell types [39], [40] and intermittent transient viremia are also frequently detected in well suppressed HIV-infected subjects on ART [39], [41], [42]. Such viral blips are thought to result from activation of latently-infected cells by antigen recognition or bystanders in a local inflammatory response [43], suggesting that LtxA treatment may be effective against these activated cells. Nevertheless, LtxA is not likely to eradicate latent HIV reservoirs in resting CD4 T cells or other cell types that do not express high levels of activated LFA-1. LtxA also may have unintended effects on uninfected LFA-1-bearing leukocytes including virus-specific T cells needed for controlling HIV infection. Moreover, HIV infection has been consistently associated with increased numbers of activated uninfected bystander CD4 and CD8 T lymphocytes [44], [45], and this may pose a serious challenge in developing LtxA for an HIV therapeutic drug as these cells are likely more susceptible to LtxA. Future studies are needed to investigate this possibility in order to fully appreciate the potential of LtxA as an anti-HIV therapeutic agent.

In summary, this study demonstrates for the first time the ability of HIV envelope gp120 to directly induce LFA-1 activation on CD4 T cells in a CD4-dependent mechanism. LFA-1 activation on target and infected CD4 T cells may enhance HIV infectivity and transmission by promoting virus binding and cell-to-cell spread, but it also increases the cell susceptibility to a bacterial toxin, LtxA that preferentially targets active LFA-1. This finding opens a possibility to exploit this LFA-1-targeting leukotoxin as an anti-HIV agent for reducing or clearing virus burden in HIV-infected subjects.

## Materials and Methods

### Cells

PBMCs from healthy donors were isolated from leukopacs, which were purchased from the New York Blood Center; these blood donors were anonymous. PBMCs from HIV-infected subjects were from whole blood; all HIV-infected subjects recruited for this study gave written informed consent. The New York University Institutional Review Board approved the use of human specimens for this study. After Ficoll-Paque Plus centrifugation, PBMCs were used directly in the experiments or were first enriched for CD4 T cells using a negative-selection magnetic bead kit (Miltenyi Biotech or StemCell Tech) as described previously [25].

### Leukotoxin (LtxA)

LtxA was purified from culture supernatants of A. *actinomycetemcomitans* strain NJ4500 as previously described [46], [47]. Protein was lyophilized in sterile vials and stored at −80°C. Samples were reconstituted in sterile distilled water and filtered through a 0.22 µm filter prior to use. When prepared in this manner, LtxA was stable for at least 6 months.

### Planar bilayer assay and microscopy

Planar bilayers were prepared as described previously [25], [26] from liposomes containing 12.5% Ni^2+^-chelating DOGS-NTA (1,2-dioleoyl-*sn*-glycero-3-[*N*(5-amino-1-carboxypentyl) iminodiacetic acid] succinyl and glycosylphosphatidylinositol (GPI)-anchored or His_12_-tagged Cy5-labeled mouse ICAM-1 (density of 200-250 molecules/µm^2^). His_6_-gp120 of HIV-1 DH12 used to reconstitute the bilayers was produced from recombinant vaccinia virus [48], labeled with Alexa Fluor 488 (Invitrogen), and applied onto the bilayers at a concentration that resulted in gp120 density of 200 to 250 molecules/µm^2^. After the flow cell containing the bilayers was warmed to 37°C, cells were injected and images collected for 1 hr on a wide-field fluorescence microscope. To test the effects of anti-gp120 mAbs, bilayers were first treated for 20 min with 20 µg/ml of each mAb (EH21, 2219, and 654). The cells were also suspended in buffer containing 20 µg/ml of the mAb before injection to the bilayers. Bilayers containing anti-CD4 and anti-CD3 antibodies were also prepared as previously described [25], [26], using monobiotinylated and Alexa Flour 488 labeled anti-CD3ε (OKT3) or anti-CD4 (OKT4) monoclonal antibodies from eBioscience. OKT3 and OKT4 were applied onto the bilayers at 250 molecules/µm^2^, a density comparable to that of gp120. The densities of these molecules were determined at each experiment by coating the same bilayer preparations onto 5 µm silica beads and analyzing the beads by flow cytometry using fluorescein calibration beads.

Multicolor fluorescence microscopy was performed on an automated microscope with an Orca-ER cooled charge-couple-devise (CCD) camera or electron multiplier CCD camera (Hamamatsu). The hardware on the microscope was controlled using Scanalytics IP-Lab software on a Dell personal computer. Solamere Technology provided integration support. Image processing and analyses were performed with IP Lab and Metamorph software. TIRF microscopy was performed either on an Olympus IX70 or a Nikon Eclipse Ti microscope with either a Hamamatsu Orca-ER CCD or an Andor iXon 897 EMCCD camera. Nikon TIRF microscopy images were flat-field corrected using ImageJ 1.45b.

### Flow cytometric analysis

Surface and intracellular staining of PBMCs was done as previously described [49]. Fluorescence-conjugated antibodies to CD3 (APC-Cy7) and CD8 (APC) were used to gate CD4 T cell population (CD3+ CD8-) studied, and FITC-conjugated anti-p24 monoclonal antibody (KC57; Coulter) was used to detect CD4 T cells with active HIV replication. Total LFA-1 expression was measured with PE-conjugated anti-CD11a mAb (BD Pharmingen), while active LFA-1 was detected with mAb NKI-L16, which was a generous gift from Drs. C. Figdor and B. Joosten (Radboud University Nijmegen Medical Centre, Nijmegen, The Netherlands) [29]. Data analyses were done with the FlowJo software (Tree Star).

### Cell viability assay

CD4 T cells (1×10^5^/well) were added to microtiter wells pre-coated with wild type, mutant, or no gp120 (10 µg/ml) and then treated with LtxA at the designated concentrations. Soluble gp120 was added to the cells at 10 µg/ml. For comparison, cells activated with anti-CD3/anti-CD28 (5 µg/ml each) or anti-CD4 (10 µg/ml) antibodies on microtiter wells were also treated with LtxA. In some experiments, cells were incubated on gp120-coated wells in the presence of anti-gp120 monoclonal antibodies (20 µg/ml), soluble CD4 (20 µg/ml), or chemokine receptor antagonists (10 µM). The culture medium contained RPMI, 10% fetal bovine serum, L-glutamine, and penicillin/streptomycin. After 20 hrs, cellular viability after LtxA treatment was determined using the CellTiter-Glo luminescent cell viability assay (Promega). Plates were read in Perkin Elmer Victor3 Multilabel Counter in the luminescence mode.

### Real-time PCR

Cells were lysed by incubation with lysis buffer (5 mM Tris [pH 8.3], 0.45% Triton X-100, 0.45% Tween 20) and proteinase K (20 mg/ml) for 1 hr at 60°C and then for 15 min at 95°C to inactivate proteinase K. Cell lysate (2 µl each) was then used in a 20 µl reaction on the Applied Biosystems 7900HT Fast Real-Time PCR System with 1X SYBR Green Supermix (SYBR Green I Dye, AmpliTaq Gold® DNA Polymerase, dNTPs with dUTP, passive reference dye, and optimized buffer components) (Applied Biosystems) and the specific primers (5 picomoles each). For measuring gag, a 115-bp fragment in the gag region was amplified using the primers SK38 (5′-ATAATCCACCTATCCCAGTAGGAGAAAT-3′) and SK39 (5′-TTTGGTCCTTGTCTTATGTCCAGAATGC-3′) (Invitrogen) [50] under the following reaction condition: 95°C for 10 min (initial denaturation) and 40 cycles of 95°C for 15 sec (denaturation) and 60°C for 1 min (primer annealing and extension). For β-actin, a 217 bp fragment was amplified using primers 5′-CTCCATCCTGGCCTCGCTGT-3′ and 5′-CACCTTCACCGTTCCAGTTT-3′ in the following reaction condition: 95°C for 10 min (initial denaturation) and 40 cycles of 95°C for 30 sec (denaturation), 55°C for 30 sec (primer annealing), 60°C for 1 min (primer extension). PCR products were quantified based on the standard curve in each experiment. The 8E5 LAV cells, each of which contains 1 copy of HIV provirus and 2 copies of β-actin gene, were used to generate the standard curves.

## Supporting Information

Figure S1 **Surface phenotype of naïve CD4 T cells enriched from PBMCs of HIV-seronegative donors.** Naïve CD4 T cells were enriched by negative selection with antibody-coated magnetic beads and then treated with fluorescent antibodies to CD3, CD4 (A), CD45RA, and CD45RO (B). C) These naive cells were also stained with mAb NKI-L16 which detects an epitope present specifically in the active state, extended conformation of LFA-1 α chain (left panel). Whole PBMCs stained with NKI-L16 are shown for comparison (right panel).
(TIF)

Figure S2 **A) The background p24 staining of CD4 T cells (CD3+ CD8-) from a HIV-seronegative donor, NG05.** This gating was used to determine positive p24 staining in the CD4 T cells of HIV-seropositive subjects. B) CD3 expression on p24+ and p24- CD4 T cell populations from HIV-infected subjects PS05 and PS07. The mfi for p24+ and p24- cells are 87 and 85 for PS05, and 74 and 61 for PS07.
(TIF)

Figure S3 **Measurement of β-actin copy numbers in HIV-infected PBMCs after LtxA treatment.** PBMCs from two viremic HIV-infected subjects (PS05 with 38,165 vRNA copies/ml and CD4 count of 814 and PS14 with 21,815 vRNA copies/ml and CD4 count of 494) were treated with LtxA (7.8 µg/ml) for 20 hrs. The β-actin copy numbers were quantified by real time PCR with the specific primers. Averages and standard deviation from 4 repeat experiments are presented.
(TIF)
