# Supplementary figures and images for: HIV Envelope gp120 Activates LFA-1 on CD4 T-Lymphocytes and Increases Cell Susceptibility to LFA-1-Targeting Leukotoxin (LtxA)
Source: PLoS One. 2011 Aug 5;6(8):e23202. doi: 10.1371/journal.pone.0023202 (PMC3151267; doi:10.1371/journal.pone.0023202)

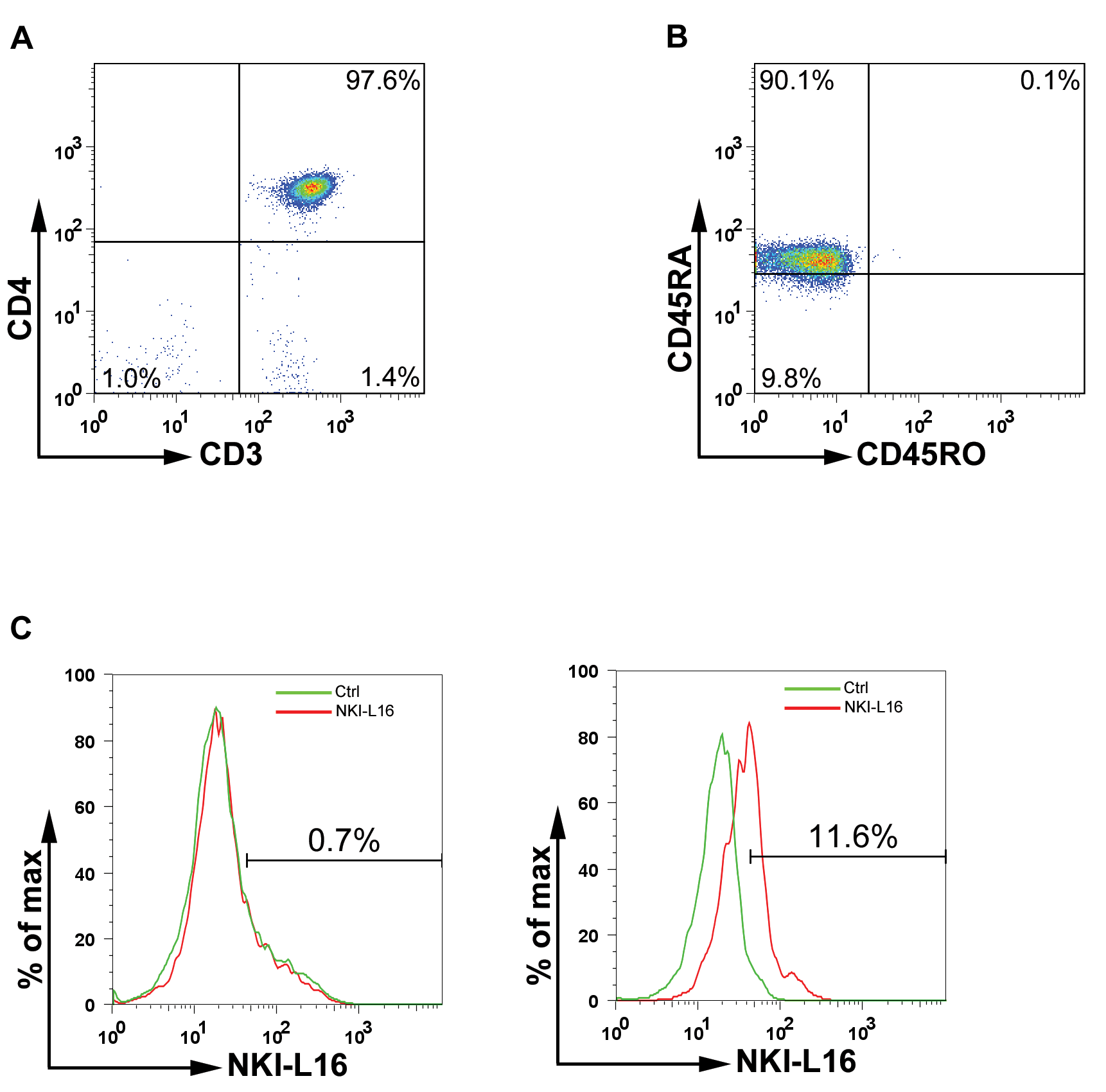

Supplement: Figure S1 — Surface phenotype of naïve CD4 T cells enriched from PBMCs of HIV-seronegative donors. Naïve CD4 T cells were enriched by negative selection with antibody-coated magnetic beads and then treated with fluorescent antibodies to CD3, CD4 (A), CD45RA, and CD45RO (B). C) These naive cells were also stained with mAb NKI-L16 which detects an epitope present specifically in the active state, extended conformation of LFA-1 α chain (left panel). Whole PBMCs stained with NKI-L16 are shown for comparison (right panel). (TIF) [file pone.0023202.s001.tif]

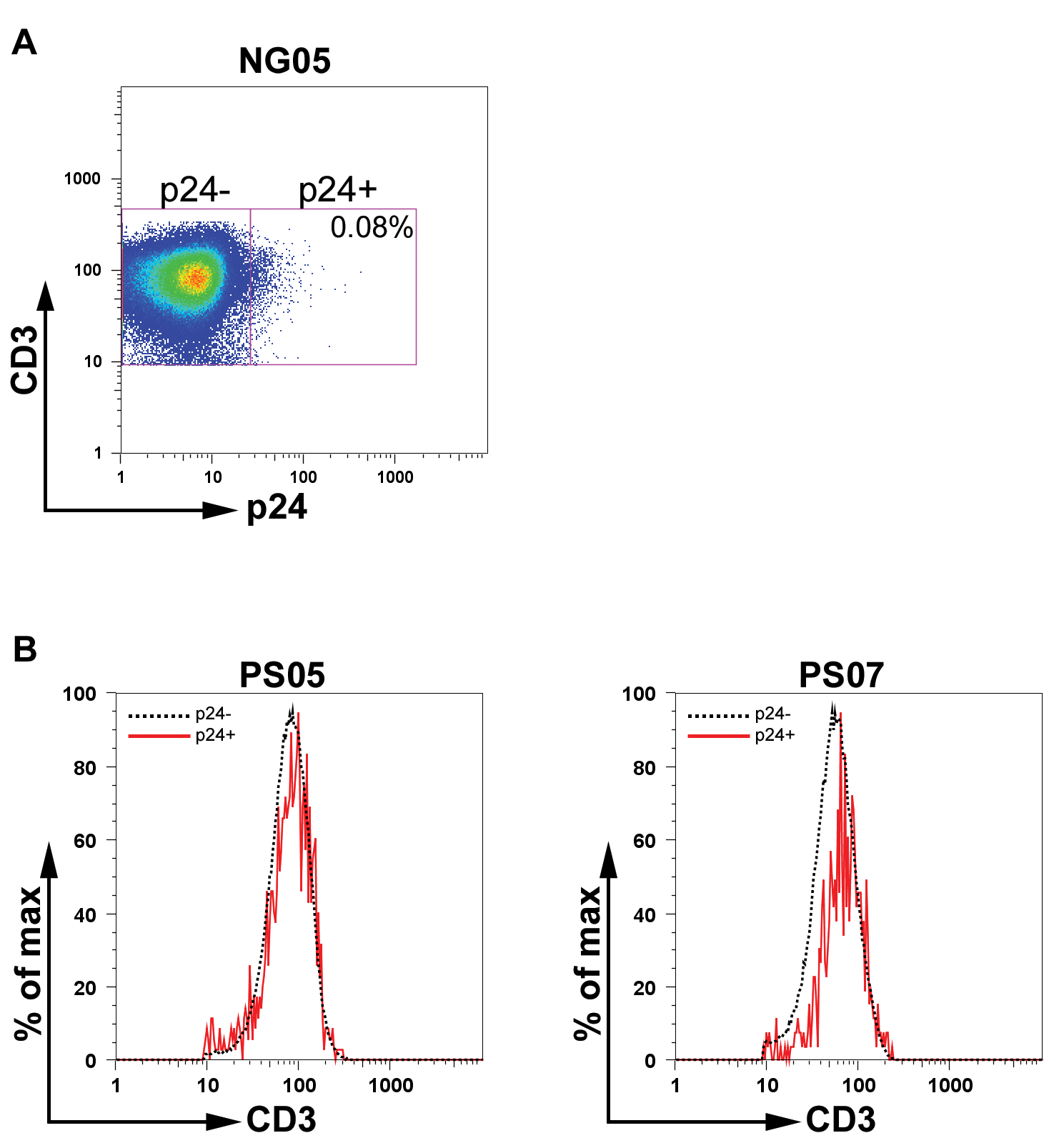

Supplement: Figure S2 — A) The background p24 staining of CD4 T cells (CD3+ CD8-) from a HIV-seronegative donor, NG05. This gating was used to determine positive p24 staining in the CD4 T cells of HIV-seropositive subjects. B) CD3 expression on p24+ and p24- CD4 T cell populations from HIV-infected subjects PS05 and PS07. The mfi for p24+ and p24- cells are 87 and 85 for PS05, and 74 and 61 for PS07. (TIF) [file pone.0023202.s002.tif]

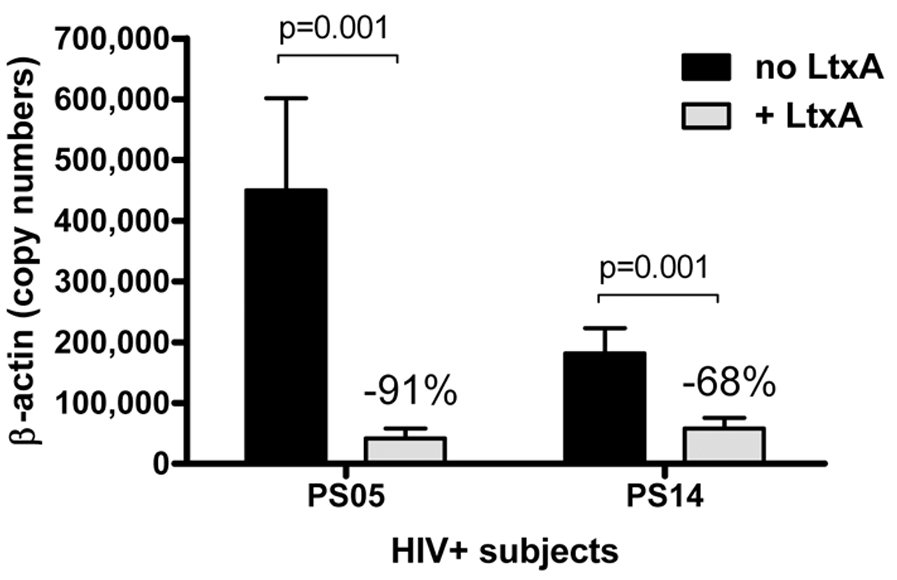

Supplement: Figure S3 — Measurement of β-actin copy numbers in HIV-infected PBMCs after LtxA treatment. PBMCs from two viremic HIV-infected subjects (PS05 with 38,165 vRNA copies/ml and CD4 count of 814 and PS14 with 21,815 vRNA copies/ml and CD4 count of 494) were treated with LtxA (7.8 µg/ml) for 20 hrs. The β-actin copy numbers were quantified by real time PCR with the specific primers. Averages and standard deviation from 4 repeat experiments are presented. (TIF) [file pone.0023202.s003.tif]
